# BART-Survival: A Bayesian machine learning approach to survival analyses in Python

**DOI:** 10.21105/joss.07213

**Published:** 2025-01-28

**Authors:** Jacob Tiegs, Julia Raykin, Ilia Rochlin

**Affiliations:** 1 Inform and Disseminate Division, Office of Public Health Data, Surveillance, and Technology, Centers for Disease Control and Prevention, Atlanta, Georgia, United States of America; 2 Metas Solutions, Atlanta, Georgia, United States of America

## Abstract

BART-Survival is a Python package that allows time-to-event (survival) analyses in discrete-time using the non-parametric machine learning algorithm, Bayesian Additive Regression Trees (BART). BART-Survival combines the performance of the BART algorithm with the complementary data and model formatting required to complete the survival analyses. The library contains a convenient application programming interface (API) that allows a simple approach when using the library for survival analyses, while maintaining capabilities for added complexity when desired. The package is intended for analysts exploring use of flexible non-parametric alternatives to traditional (semi-)parametric survival analyses.

## Statement of need

Survival analyses are a cornerstone of public health and clinical research in such diverse fields as cancer, cardiovascular disease, and infectious diseases (Altman & Bland, 1998; Bradburn et al., 2003). Traditional parametric and semi-parametric statistical methods, such as the Cox proportional hazards model, are commonly employed for survival analyses (Cox, 1972). However, these methods have several limitations, particularly when applied to complex data. One major issue is the need for restrictive assumptions, such as proportional hazards and predefined functional forms, which may not hold true in complex, real-world healthcare data (Harrell, 2015; Ishwaran et al., 2008). Additionally, these methods often struggle with high-dimensional datasets, leading to problems with over-fitting, multi-collinearity, and dealing with complex interactions (Ishwaran et al., 2008; Joffe et al., 2013).

More recently, non-parametric machine learning approaches have been introduced to address the limitations of the traditional methods (Harrell, 2015; Ishwaran et al., 2008). BART is a one such approach that has demonstrated exceptional performance across a variety of analytic settings, typically outperforming the traditional methods in terms of predictive accuracy. BART’s performance is linked to it ability to flexibly model complex non-linear and variable interactions within the data, while being inherently regularized to reduce issues of over-fitting. The BART method is fully non-parametric and can adaptively model data complexities without prior knowledge or specification of a particular functional form. Finally, the method is generally accepted as being a user-friendly machine learning approach, as it typically requires minimal hyperparameter tuning and the outcomes can be easier to interpret than those produced by other similar methods (Chipman et al., 2010; R. Sparapani et al., 2021; R. A. Sparapani et al., 2016).

Currently, the only BART survival algorithm readily available exists as part of the BART R package, which contains a library of various BART-based approaches in addition to a BART survival analysis application (R. Sparapani et al., 2021; R. A. Sparapani et al., 2016). The BART-Survival package described here combines the survival analysis approach outlined in the BART R package with the foundational Python-based probabilistic programming language library, PyMC (Abril-Pla et al., 2023), and the accompanying BART algorithm from the PyMC-BART library (Quiroga et al., 2023). Our aim in developing BART-Survival is to provide accessibility to the BART survival algorithm within the Python programming language. This contribution is beneficial for analysts when Python is the preferred programming language, the analytic workflow is Python-based, or when the R language is unavailable for analyses.

The need for a complete BART-Survival python package is given by the simple fact that the BART survival algorithm is non-trivial to implement. Both the required data transformations and the internal model definition requires precise implementations to ensure generation of accurate survival models. Our BART-Survival library provides accessibility to these precise methods while removing the technical barriers that would limit user adoption of the BART survival approach.

More specifically, the BART-Survival library abstracts away the complexities of generating the proper training and inference datasets, which are conceptually complex and prone to being specified incorrectly if implemented from scratch. Similarly, the BART-Survival library provides a pre-specified internal Bayesian model using the PyMC probabilistic programming language. This pre-specified model is primarily accessed through the BART-Survival API removing the requirement for users to have more than a cursory knowledge of the PyMC or PyMC-BART libraries. Since the BART-Survival package is intended for students and professional in the public health and clinical fields, it is expected that users of the BART-Survival library will not have extensive programming expertise, adding to the need for a fully self-contained and accessible approach.

In summary, the BART-Survival package provides a simple and accessible approach to implementing the BART survival algorithm. The provided approach can be beneficial for users who are looking for non-parametric alternatives to traditional (semi-)parametric survival analysis. The BART survival algorithm can be especially useful in large, complex healthcare data, where machine learning methods can demonstrate improved performance over the traditional methods.

## Conclusion

BART-Survival provides the computational methods required for completing non-parametric discrete-time survival analysis. This approach can have several advantages over alternative survival methods. These advantages include capabilities to incorporate non-linear and interaction effects into the model, naturally ability to regularize the model (which reduces the risk of over-fitting) and of being robust to issues of multi-collinearity. The BART-Survival approach is especially useful when the assumptions of alternative survival methods are violated.

Our BART-Survival algorithm has been tested in a rigorous simulation study, with additional applications to real-world data. While the manuscript for this work is currently under development, the results indicate similar performance as the the R-based BART survival method across settings of varied complexity. Both methods demonstrate the previously describe advantages over other survival approaches (such as Cox Proportional Hazard Models) when the relationships within the data becomes more complex or assumptions of the these other models are violated. A comparison of the R-based method and our BART-Survival algorithm is included in the examples folder of the Github repository.

Our library provides a convenient API for completing discrete-time survival analysis, along with the functionality to customize the methodology as needed. The associated API documentation can be found here, along with the associated Github repository BART-Survival. An extended review of the methods is additionally provided within the documentation and can be found here.
